# The complete chloroplast genome sequence of *Melochia corchorifolia* Linnaeus, 1753 (Sterculiaceae)

**DOI:** 10.1080/23802359.2024.2305711

**Published:** 2024-01-24

**Authors:** Wen Wang, Xingya Wang

**Affiliations:** School of Pharmaceutical Sciences, Zhejiang Chinese Medical University, Hangzhou, China

**Keywords:** *Melochia corchorifolia* Linn, Sterculiaceae, chloroplast genome, phylogenetic analysis

## Abstract

*Melochia corchorifolia* Linnaeus, 1753, is a weedy tropical plant of the Sterculiaceae family and has medicinal value. We sequenced the complete chloroplast genome of *M. corchorifolia* using Illumina high-throughput sequencing and examined phylogenetic relationships with closely related species. The assembled chloroplast genome of *M. corchorifolia* was 163,693 bp long and contained a pair of inverted repeats of 29,729 bp, separated by a large single-copy sequence of 84,350 bp and a small single-copy region of 19,885 bp. A total of 136 genes were annotated across the entire chloroplast genome, including 37 transfer RNA, 8 ribosomal RNA, and 91 protein-coding genes. The GC content of the complete cp genome was 37.27%. The phylogenetic tree indicated that *M. corchorifolia* is closely related to *Melochia pyramidata* (Malvaceae). These results would provide useful information for future phylogenetic, taxonomic, and evolutionary studies on Sterculiaceae and Malvaceae.

## Introduction

*Melochia corchorifolia* Linnaeus, 1753, commonly known as chocolate weed, is a semi-shrub herbaceous plant that has been placed in the family Sterculiaceae but also in the closely related family Malvaceae (Wilkie et al. 2006; Xu et al. 2013; Li et al. 2022). *M. corchorifolia* is commonly found in wastelands worldwide, and it has been traditionally used as a remedy for itching, rashes, ulcers, and pain of the head, stomach, and chest (Rao et al. 2013). Laboratory studies have shown that *M. corchorifolia* extracts exhibit hepatoprotective, antioxidant, and anti-pest effects *in vitro* (Pavunraj et al. 2012; Rao et al. 2013). *M. corchorifolia* extracts can inhibit melanogenesis in B16F10 melanoma cells because of their ability to suppress the expression of tyrosinase- and microphthalmia-associated transcription factors (Yuan et al. 2020). These properties take effect through the potent bioactive components of *M. corchorifolia*, including cyclopeptide alkaloids, melofoline, franganine, flavonoids, apigenin, kaempferol, and quercetin (Rajendra et al. 1986, 1991; Trlpath et al. 2010; Rao et al. 2013). Current research on *M. corchorifolia* has focused on its chemical composition, biological functions, and protective effects against plant diseases (Rao et al. 2013; Lu et al. 2016; Pavunraj et al. 2021; Yu et al. 2022). The chloroplast genome has long been a focus of research on plant molecular evolution and systematics because of its small size, high copy number, and conserved sequences among species (Clegg et al. 1994; Jo et al. 2022). It is important to resolve the phylogenetic relationships of species and their evolution; however, only a few complete chloroplast genome sequences of species in the genus *Melochia* have been reported. Here, whole-chloroplast genome sequencing of *M. corchorifolia* is reported for the first time, which will provide important information on the genetic resources and phylogenetic relationships of the genus *Melochia* and the family Sterculiaceae.

## Materials and methods

**Figure 1.**
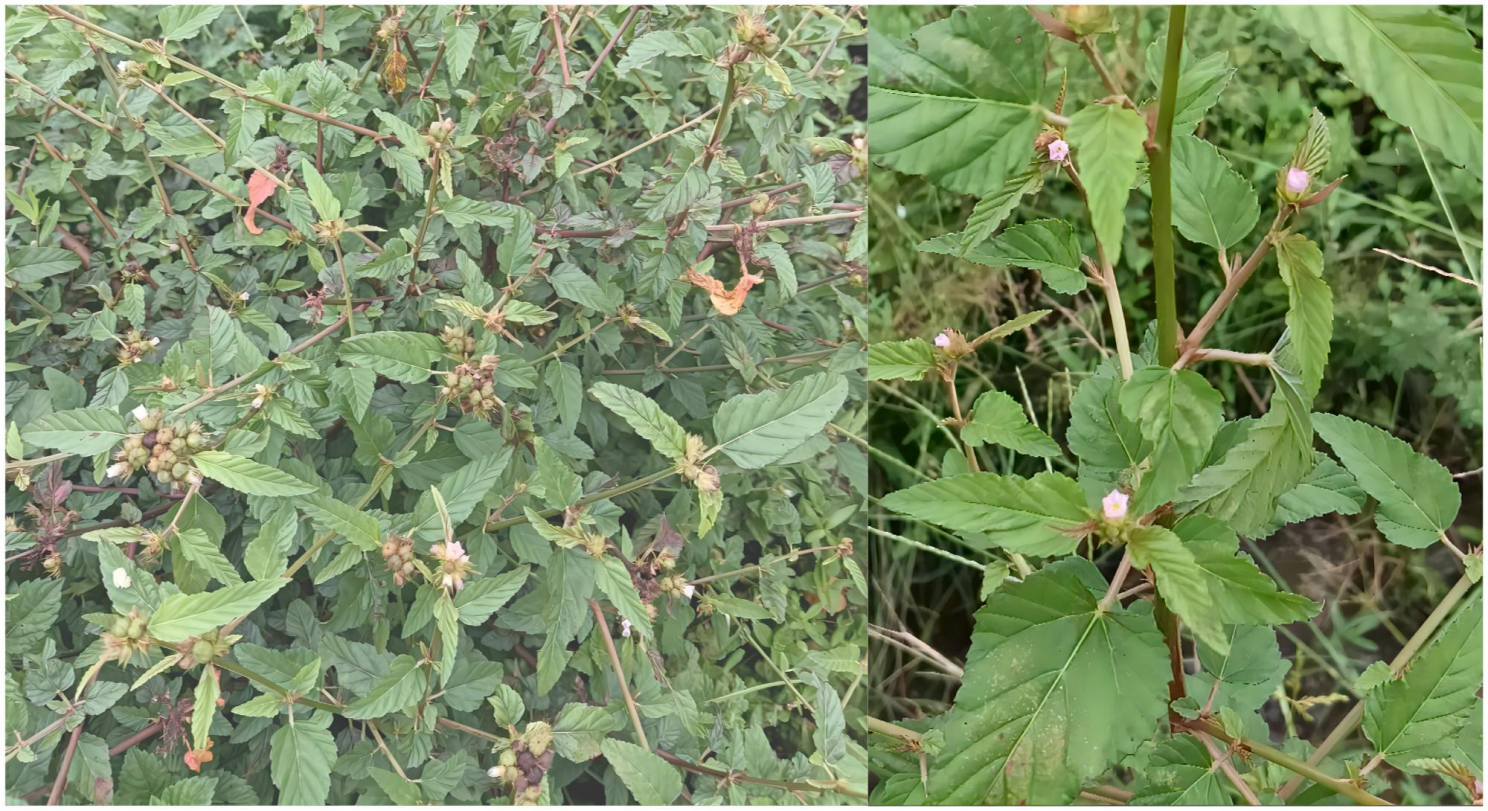
Reference image of *M. corchorifolia*. This image was taken by Wen Wang in the Baicao Garden of Zhejiang Chinese Medical University. The plant height is 1–6 ft; branches are yellowish-brown; leaves are variable in shape, simple, petiolate, ovate to lanceolate in outline, and doubly serrate. Simple hairs occur along the major veins on the lower surface of the leaf; the leaf base is round or heart-shaped, and stipules are bar-shaped; flowers are produced in terminal head-like cymes and are arranged in umbrella inflorescences or clusters of umbrella inflorescences apically or axillarily, with petals that can change from white to reddish.

Fresh and healthy leaves of *M. corchorifolia* were collected in Zhejiang (119°89′30.64″ E, 30°8′42″ N; Figure 1). Voucher specimens and samples (DNA) were stored at the Herbarium of the College of Pharmaceutical Sciences, Zhejiang Chinese Medical University (https://www.zcmu.edu.cn/, contact: Xingya Wang, xywang@zcmu.edu.cn) under voucher number ZCMU4C507. Total genomic DNA was extracted from *M. corchorifolia* leaves using a Fast Plant DNA Extraction Kit (DP305; Tiangen Biotech, Beijing, China). Total DNA was extracted, fragmented by ultrasonication, and purified. Sequencing libraries were constructed with an insert size of 335 bp and sequenced on an Illumina NovaSeq 6000 platform (Illumina, San Diego, CA, USA) at Biogenome Sequencing Corporation (Nanjing, China).

After adapter removal, raw data were obtained and analyzed using fastp v0.20.0 (https://github.com/OpenGene/fastp) (Chen et al. 2018), and high-quality reads (6.26 G) (supplemental Table S1) were assembled using SPAdes v3.10.1 (http://cab.spbu.ru/software/spades/) (Bankevich et al. 2012) and SSPACE v2.1.1 (https://sourceforge.net/projects/gapfiller/) (Boetzer and Pirovano 2012), with *Gossypium nandewarense* as the reference (GenBank accession number: NC_039568.1) (Wu et al. 2018). We then verified the accuracy of the assembly by mapping clean reads back to the assembled complete chloroplast genome sequence using Geneious Prime 2022.2 (https://www.geneious.com/) to assess the depth of coverage (supplemental Figure S1).

The assembled sequences of *M. corchorifolia* were annotated using Prodigal v2.6.3 (https://www.github.com/hyattpd/Prodigal), Hmmer v3.1b2 (http://www.hmmer.org/) (Eddy 2009), and Aragorn v1.2.38 (Laslett and Canback 2004) and were screened using BLAST (Johnson et al. 2008) to remove incorrect and redundant annotation. A cis/trans-splicing gene map was produced using CPGview software (http://www.1 kmpg.cn/cpgview) (Liu et al. 2023), which is shown in supplemental Figure S2. A circular map of the complete cp genome of *M. corchorifolia* was produced using OGDRAW (https://chlorobox.mpimp-golm.mpg.de/OGDraw.html) (Greiner et al. 2019).

Finally, we downloaded the complete chloroplast genome sequences of 11 species of the families Sterculiaceae and Malvaceae from the NCBI Nucleic Acid Resource Database, with *Elaeocarpus hainanensis* of the Elaeocarpaceae as an outgroup. We compared the genome sequences of *M. corchorifolia* and other species using MAFFT v7.4 (Katoh and Standley 2013). A maximum likelihood tree with 1000 bootstrap repeats was established using MEGA 11 and a General Time Reversible model (Tamura et al. 2021).

## Results

**Figure 2.**
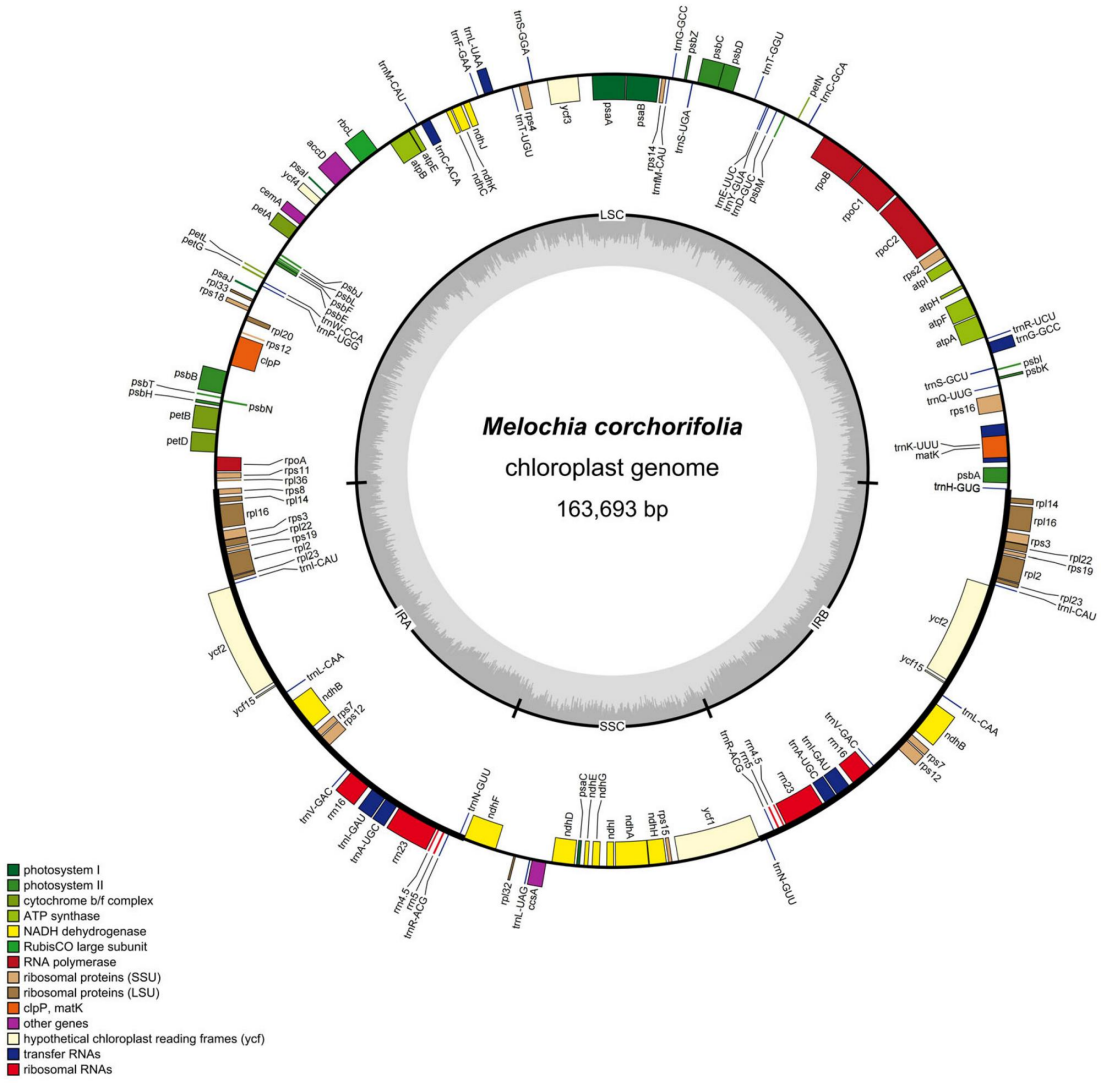
Map of the circular chloroplast genome of *M. corchorifolia*. Various functional genes were annotated across the assembled genome, as indicated by different colors. Genes outside the circle were transcribed in a counterclockwise direction, whereas genes inside the circle were transcribed in a clockwise direction. The circular chloroplast genome consisted of the inverted repeats, the small single-copy, and the large single-copy sequences. From the outside toward the Central grey inner circle, dark grey represents the GC content, and light grey represents the at content.

The chloroplast genome structure of *M. corchorifolia* (Figure 2), similar to that of most plants, was found to be a circular molecule with a length of 163,693 bp and a typical quadripartite structure. It contained a pair of inverted repeats that were separated by large and small single-copy regions. The lengths of inverted repeats (IRs), large single-copy (LSC), and small single-copy (SSC) sequences were 29,729, 84,350, and 19,885 bp, respectively. The total GC content of the complete chloroplast genome was 37.27%; this was 41.8% for IR, 35.33% for LSC, and 31.94% for SSC sequences. A total of 136 genes were annotated across the entire chloroplast genome, including 8 ribosomal RNA (rRNA), 37 transfer RNA (tRNA), and 91 protein-coding genes, involved in photosynthesis and factors associated with their expression and assembly. Most annotated genes occurred as single copies, whereas 20 genes occurred in two copies, including four rRNAs (*rrn16*, *rrn23*, *rrn4.5*, and *rrn5*), six tRNAs (*trn*A-UGC, *trn*I-CAU, *trn*I-GAU, *trn*L-CAA, *trn*N-GUU, *trn*R-ACG, and *trn*V-GAC), and ten protein-coding genes (*ndhB*, *rpl14*, *rpl16*, *rpl2*, *rpl22*, *rpl23*, *rps12*, *rps19*, *rps3*, and *rps7*). Five of these genes (*trn*A-UGC, *trn*I-GAU, *ndhB*, *rpl16*, and *rpl2*) contained one intron, and one gene (*rps12*) had two introns.

**Figure 3.**
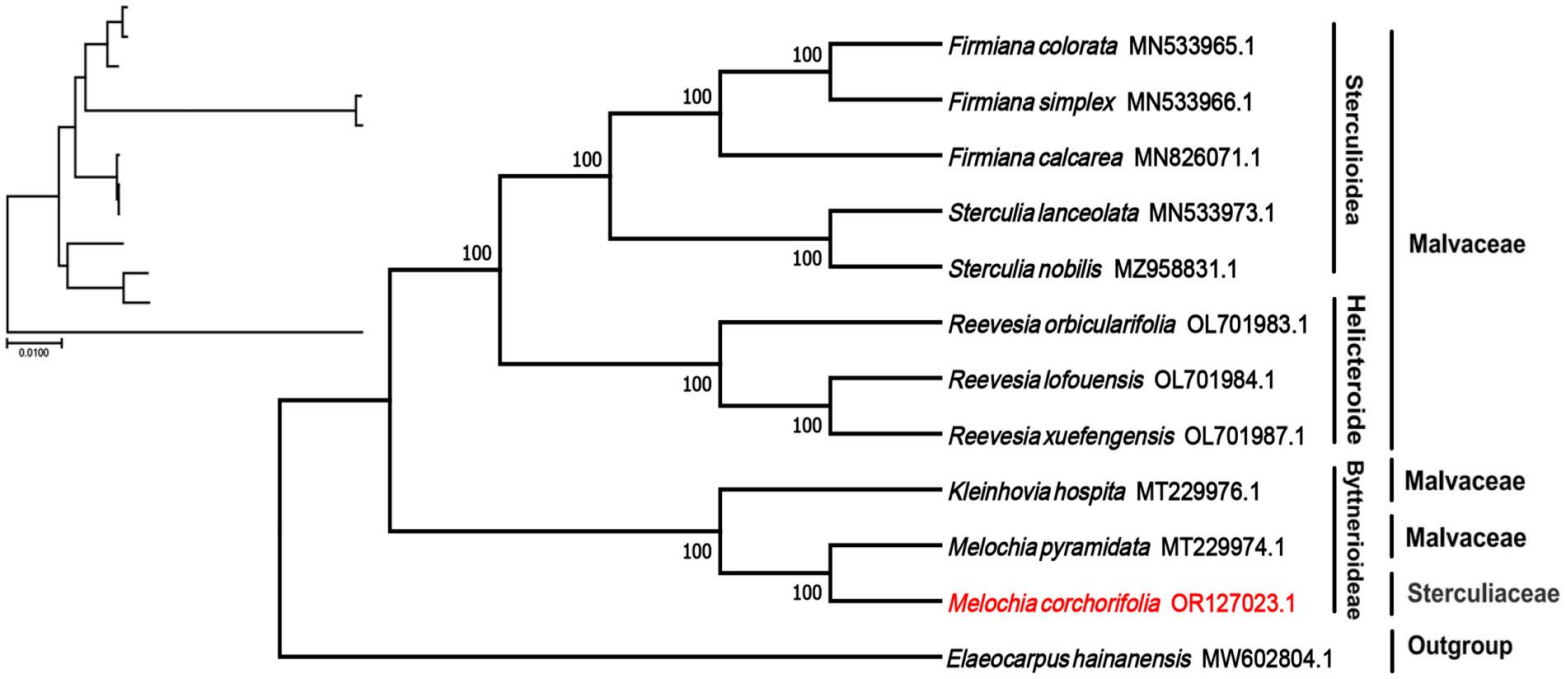
Phylogenetic tree of the complete chloroplast genome of *M. corchorifolia* with bootstrap values at each node. Sequences of the other species used to make the phylogenetic tree: *Melochia corchorifolia* (OR127023.1; this study), *Melochia pyramidata* (MT229974.1; unpublished), *Kleinhovia hospita* (MT229976.1; unpublished), *Reevesia orbicularifolia* (OL701983.1; unpublished), *Reevesia lofouensis* (OL701984.1; unpublished), *Reevesia xuefengensis* (OL701987.1; unpublished), *Firmiana calcarea* (MN826071.1; Lu and Luo 2022), *Firmiana colorata* (MN533965.1; Lu and Luo 2022), *Firmiana simplex* (MN533966.1; Lu and Luo 2022), *Sterculia lanceolata* (MN533973.1; unpublished), and *Sterculia nobilis* MZ958831.1; Lu and Luo 2022); *Elaeocarpus hainanensis* (MW602804.1; unpublished) was used as outgroup.

The phylogenetic tree analysis revealed that *M. corchorifolia* and *M. pyramidata* were sister taxa and were closely related with high bootstrap support (100%), and both species formed a clade with *Kleinhovia hospita* (Malvaceae) (Figure 3). The subfamily name was also included in the phylogenetic tree, which further confirmed that species in Sterculiaceae and Malvaceae are closely related and circumscription is not fixed. The complete chloroplast genome sequence of *M. corchorifolia* with its annotations, was deposited in the NCBI GenBank database (accession number OR127023.1).

## Discussion and conclusion

In the current study, we produced a complete 163,693-bp chloroplast genome sequence of *M. corchorifolia*, which encodes 136 genes (8 rRNA, 37 tRNA, and 91 protein-coding genes). Phylogenetic analysis revealed that *M. corchorifolia* is a sister species to *M. pyramidata*, which has 129 genes (8 rRNA, 37 tRNA, and 84 protein-coding genes). It is well known that the taxa formerly classified in the Sterculiaceae are included in the subfamilies Byttnerioideae, Dombeyoideae, Helicteroideae, and Sterculioideae of Malvaceae sensu lato, which are all closely related with each other (Wilkie et al. 2006; Péchon and Gigord 2014); however, the phylogenetic relationships among these subfamilies are not well resolved. These observations were consistent with our phylogenetic tree. For example, *Frimiana* is a small genus within the subfamily Sterculioideae of the Malvaceae (Lu and Luo 2022), which is not traditionally circumscribed in the Sterculiaceae family. *M. corchorifolia* and *M. pyramidata* belong to the same subfamily, Byttnerioideae, which belongs to the Sterculiaceae or Malvaceae family. Consistent with previous studies, the total length, overall gene order, and GC content of the entire chloroplast genome of the studied species were similar to those of other Sterculiaceae and Malvaceae species (Wang et al. 2018; Quan et al. 2019; Lu and Luo 2022). Further studies are needed to resolve the taxonomic discrepancies and biogeographical origins of Malvaceae and Sterculiaceae subfamilies. In conclusion, our study provides important information on the genetic resources and phylogenetic relationships of the genus *Melochia* and may contribute to a better resolution of evolutionary relationships within the phylogenetic clades of Sterculiaceae and Malvaceae.

## Supplementary Material

Supplemental Material

Supplemental Material

## Data Availability

The sequence information of the complete chloroplast genome assembly of *M. corchorifolia* is available on the NCBI website (https://www.ncbi.nlm.nih.gov/) with the accession number OR127023.1. BioProject, Bio-Sample, and SRA are PRJNA1000299, SAMN36767426, and SRR25460835, respectively.
