# Supplementary material for: The complete chloroplast genome sequence of Melochia corchorifolia Linnaeus, 1753 (Sterculiaceae)
Source: Mitochondrial DNA B Resour. 2024 Jan 24;9(1):153–7. doi: 10.1080/23802359.2024.2305711 (PMC10810621; doi:10.1080/23802359.2024.2305711)
Supplement: Supplemental Material [file TMDN_A_2305711_SM1494.doc]

**The complete chloroplast genome sequence of** ***Melochia corchorifolia* L.（Sterculiaceae）**

Wen Wang and Xingya Wang^*^

School of pharmaceutical sciences, Zhejiang Chinese Medical University, Hangzhou, China

*: Corresponding author: Xingya Wang, xywang@zcmu.edu.cn, School of Pharmaceutical Sciences, Zhejiang Chinese Medical University, 311400, Hangzhou, China

Table S1. The statistics of sequencing data

| Sample ID | ReadSum | BaseSum | GC (%) | Q20 (%) | Q30 (%) |
| --- | --- | --- | --- | --- | --- |
| *Melochia corchorifolia* | 20858956 | 6257686800 | 38.66 | 95.91 | 90.00 |


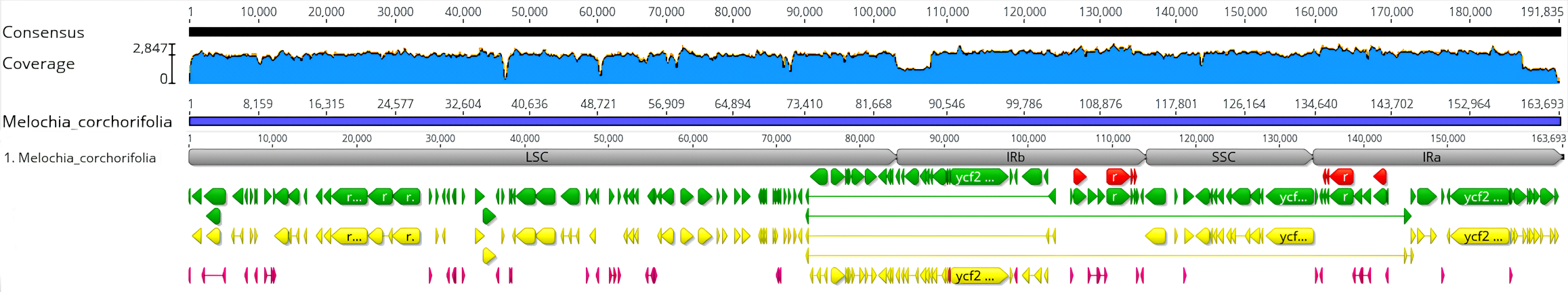


Figure S1. The overall coverage depth of chloroplast genome assembly of Melochia *corchorifolia.* The height of the blue graph indicates the number of sequences at each location. Gray bars, four regions of the genome (LSC, SSC, and two IRs); short arrows, annotation types: red (rRNA), green (gene), yellow (CDS), and pink (tRNA).

**(A)**


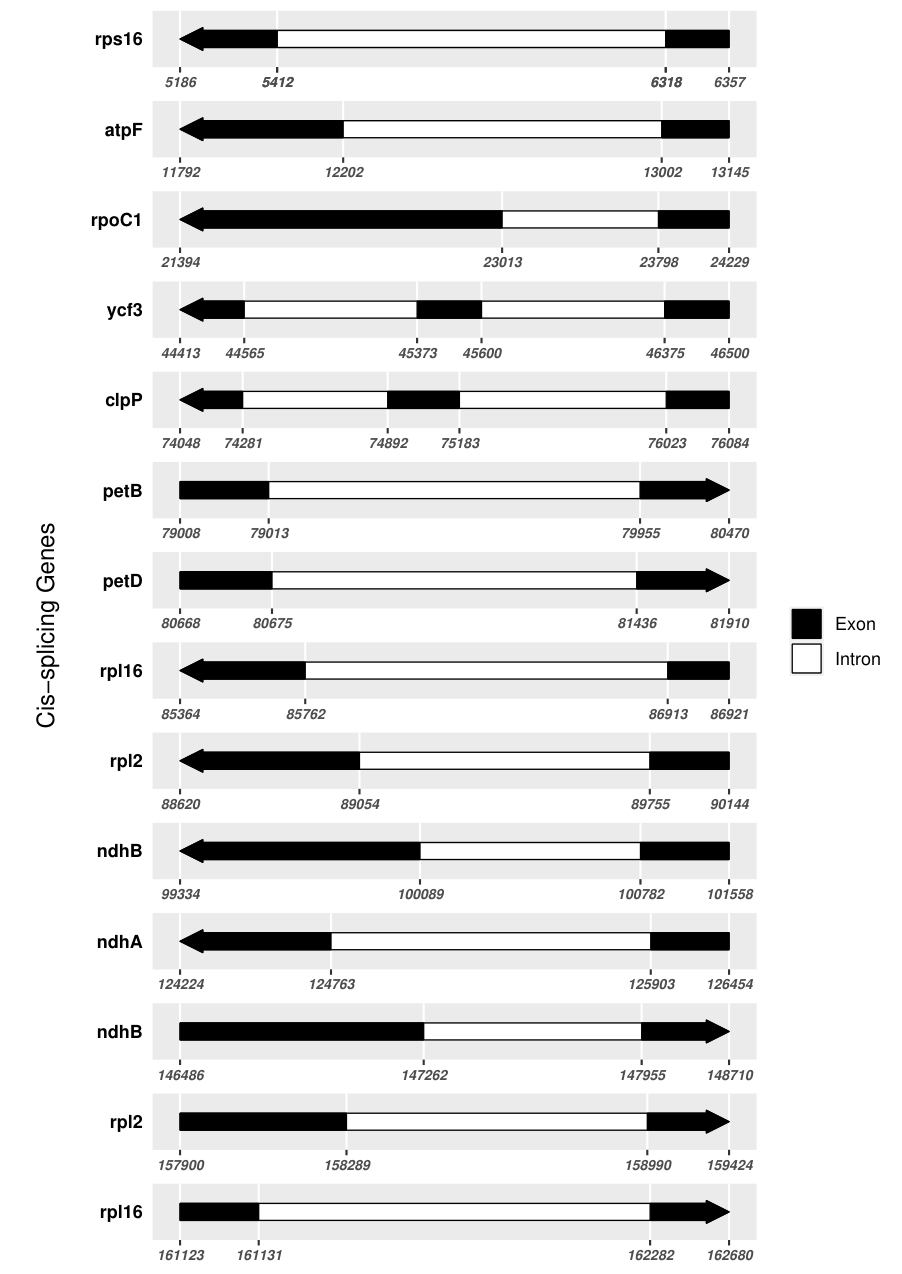

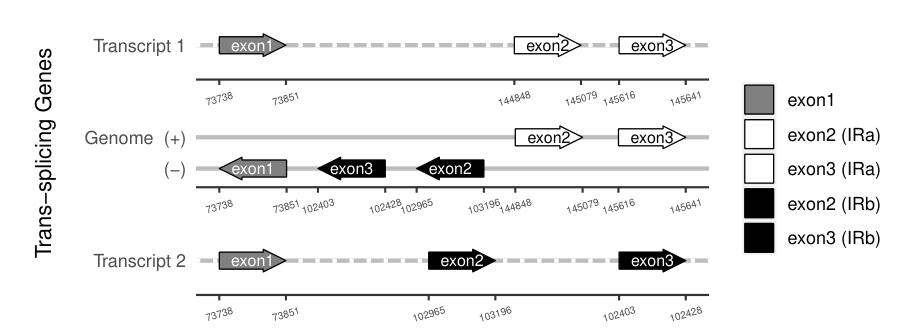


**(B)**

Figure S2. Schematic map of the cis-splicing gene and trans-splicing genes in the chloroplast genome assembly of *Melochia corchorifolia*. (A) The map of the cis-splicing genes. The exons are shown in black; the introns are shown in white. The arrow indicates the sense direction of the gene. (B) The map of the trans-splicing genes rps12. The gene names are shown on the left, and the gene structures are on the right. It has three unique exons. Two exons are duplicated as they are located in the IR regions. The map was generated using CPGview.
