# Supplementary material for: The complete chloroplast genome sequence of Melochia corchorifolia Linnaeus, 1753 (Sterculiaceae)
Source: Mitochondrial DNA B Resour. 2024 Jan 24;9(1):153–7. doi: 10.1080/23802359.2024.2305711 (PMC10810621; doi:10.1080/23802359.2024.2305711)
Supplement: Supplemental Material [file TMDN_A_2305711_SM1433.pdf]

# CERTIFICATE OF ENGLISH EDITING

This document certifies that the paper listed below has been edited to ensure that the language is clear and free of errors. The logical presentation of ideas and the structure of the paper were also checked during the editing process. The edit was performed by professional editors at Editage, a division of Cactus Communications, in cooperation with Taylor & Francis Group. The intent of the author's message was not altered in any way during the editing process. The quality of the edit has been guaranteed, with the assumption that our suggested changes have been accepted and have not been further altered without the knowledge of our editors.

## Title

The complete chloroplast genome sequence of Melochia corchorifolia Linnaeus, 1753 (Sterculiaceae)

## Authors

Xingya Wang

## Order No.

DOMTC_1

EDITINGSERVICES
Supporting Taylor & Francis authors

Signature

Prabh Grewal,
Senior Vice President,
Editage

Date of Issue
**December 15, 2023**

INFORMATION SECURITY MANAGEMENT SYSTEM DNV·GL ISO/IEC 27001
editage

**Taylor & Francis Editing Services**

www.tandfeditingservices.com
support@tandfeditingservices.com
